# Pilot Testing of an mHealth App for Tobacco Cessation in People Living With HIV: Protocol for a Pilot Randomized Controlled Trial

**DOI:** 10.2196/49558

**Published:** 2023-10-19

**Authors:** Maeve Brin, Paul Trujillo, Haomiao Jia, Patricia Cioe, Ming-Chun Huang, Huan Chen, Xiaoye Qian, Wenyao Xu, Rebecca Schnall

**Affiliations:** 1 Columbia University School of Nursing New York City, NY United States; 2 Brown University School of Public Health Providence, RI United States; 3 Case Western Reserve University School of Engineering Cleveland, OH United States; 4 Department of Computer Science and Engineering University at Buffalo, the State University of New York Buffalo, NY United States

**Keywords:** addict, addiction, app, application, applications, apps, cessation, cigar, cigarette, cigarettes, HIV, mHealth, mobile health, quit, quitting, randomized controlled trial, RCT, smartwatch, smoker, smoking cessation, smoking, tobacco

## Abstract

**Background:**

An estimated 40% of people living with HIV smoke cigarettes. Although smoking rates in the United States have been declining in recent years, people living with HIV continue to smoke cigarettes at twice the rate of the general population. Mobile health (mHealth) technology is an effective tool for people living with a chronic illness, such as HIV, as currently 84% of households in the United States report that they have a smartphone. Although many studies have used mHealth interventions for smoking cessation, few studies have recruited people living with HIV who smoke.

**Objective:**

The objective of the pilot randomized controlled trial (RCT) is to examine the feasibility, acceptability, and preliminary efficacy of the Sense2Quit App as a tool for people living with HIV who are motivated to quit smoking.

**Methods:**

The Sense2Quit study is a 2-arm RCT for people living with HIV who smoke cigarettes (n=60). Participants are randomized to either the active intervention condition, which consists of an 8-week supply of nicotine replacement therapy, standard smoking cessation counseling, and access to the Sense2Quit mobile app and smartwatch, or the control condition, which consists of standard smoking cessation counseling and a referral to the New York State Smokers’ Quitline. The Sense2Quit app is a mobile app connected through Bluetooth to a smartwatch that tracks smoking gestures and distinguishes them from other everyday hand movements. In the Sense2Quit app, participants can view their smoking trends, which are recorded through their use of the smartwatch, including how often or how much they smoke and the amount of money that they are spending on cigarettes, watch videos with quitting tips, information, and distractions, play games, set reminders, and communicate with a study team member.

**Results:**

Enrollment of study participants began in March 2023 and is expected to end in October 2023. All data collection is expected to be completed by the end of January 2024. This RCT will test the difference in outcomes between the control and intervention arms. The primary outcome will be the percentage of participants with biochemically verified 7-day point prevalence smoking or tobacco abstinence at their 12-week follow-up. Results from this pilot study will be disseminated to the research community following the completion of all data collection.

**Conclusions:**

The Sense2Quit study leverages mHealth so that it can help smokers improve their efforts at smoking cessation. Our research has the potential to not only increase quitting rates among people living with HIV who may need a prolonged, tailored intervention but also inform further development of mHealth for people living with HIV. This mHealth study will contribute significant findings to the greater mHealth research community, providing evidence as to how mHealth should be developed and tested among the target population.

**Trial Registration:**

ClinicalTrials.gov NCT05609032; https://clinicaltrials.gov/study/NCT05609032

**International Registered Report Identifier (IRRID):**

DERR1-10.2196/49558

## Introduction

### Overview

An estimated 40% of people living with HIV smoke cigarettes [1]. Smoking leads to many health problems among people living with HIV, including cardiovascular disease, pulmonary infections, bacterial pneumonia, chronic obstructive pulmonary disease, and cancer [2]. Lung cancer is the leading cause of death among people living with HIV taking antiretroviral therapy (ART) in the United States [1,3]. Although smoking rates in the United States have been declining in recent years, people living with HIV continue to smoke at twice the rate of the general population [3,4]. Quitting smoking can significantly decrease the risk of acquiring serious illnesses [2].

Mobile health (mHealth) technology is an effective tool for people living with a chronic illness, such as HIV. Currently, 84% of households in the United States report that they have a smartphone [5]. Due to the high rate of smartphone ownership and associated tech literacy, mHealth apps are an accessible and affordable means of delivering health care [6].

Although many studies have used mHealth interventions for smoking cessation [7-9], few studies have recruited people living with HIV [10-12]. As such, this study’s team created the Sense2Quit app, using the Information Systems Research (ISR) framework [13], for people living with HIV who want to quit smoking. Through an iterative process with people living with HIV who were current or former smokers, as well as usability experts, we identified major themes associated with quitting smoking and developed and evaluated the Sense2Quit app to be used in our randomized controlled trial (RCT) starting in March 2023 [14,15].

### Study Objective

The objective of this pilot RCT is to examine the feasibility, acceptability, and preliminary efficacy of the Sense2Quit app as a tool for people living with HIV who are motivated to quit smoking. This paper details the procedures that will take place during the Sense2Quit RCT.

## Methods

### Ethical Considerations

All study procedures were reviewed and approved by the Columbia University institutional review board (protocol number AAAT7031). Study participants will provide written informed consent and HIPAA (Health Insurance Portability and Accountability Act) authorization before enrolling in the study. Consent forms inform participants about (1) why the study is being carried out, (2) the things they will be asked to do if they are part of the study, (3) any known risks involved, (4) any potential benefit, (5) options other than taking part in this study that they have, and (6) the way their health information will be used and shared for research purposes. The study staff explains all procedures that will occur throughout the study and answers any participant questions before obtaining the participant’s informed consent.

Confidential participant information will be assigned a code number and separated from any name or information that is identifiable. The research file that links a participant name to the code number will be kept on a password-protected computer, and only the investigator and authorized study staff will have access to the file. Any results disseminated from the study will be deidentified.

All participants will receive US $40 after completing baseline procedures, US $50 after completing their 4-week follow-up, and US $60 after completing their 12-week follow-up. All tokens of appreciation will be presented in the form of a Bank of America pay card.

### Design

The Sense2Quit study is a 2-arm RCT among people living with HIV who are motivated to quit smoking. Sense2Quit is a 12-week pilot study to determine the feasibility and acceptability of the intervention. This study duration is comparable to other small pilot feasibility and acceptability studies [16-21]. Participants are randomized to either the active intervention condition, which consists of an 8-week supply of nicotine replacement therapy (NRT), standard smoking cessation counseling, and access to the Sense2Quit mobile app and smartwatch, or the control condition, which consists of standard smoking cessation counseling and a discussion about enrolling in the New York State (NYS) Smokers’ Quitline, whereby the study team encourages the participant to contact the Quitline. Those who contact the NYS Quitline are eligible to receive an NRT supply, smoking cessation counseling, and other benefits, depending on their zip code [22]. Participants in the control arm do not receive NRT as part of the study because the study team determined that offering NRT would not be considered a true standard of care. Intervention participants will self-report NRT use on the follow-up visit surveys. Control participants will receive a sheet with information about the NYS Quitline and will self-report whether or not they contacted the Quitline during check-in calls with the study team. Participants in both conditions are scheduled for a baseline visit, a 4-week follow-up, and a 12-week follow-up. Participants are informed that the day of their baseline visit is their quit date; however, they are given the option to set their quit date on an established date within 1 week following their baseline appointment. Study team members will call participants in both arms to check in on a weekly or biweekly basis about their quitting process. The differences in study arms are presented in Table 1.

**Table 1:** Intervention versus control study procedures.

Study procedure			Baseline		4-week follow-up		12-week follow-up
**Intervention**							
	Survey	✓		✓		✓	
	Smoking cessation counseling	✓					
	eCO^a^ monitoring	✓		✓		✓	
	8-week supply of combination NRT^b^	✓					
	Sense2Quit app and smartwatch	✓					
**Control**							
	Survey	✓		✓		✓	
	Smoking cessation counseling	✓					
	eCO monitoring	✓		✓		✓	
	NYS^c^ Smokers’ Quitline referral	✓					

^a^eCO: exhaled carbon monoxide.

^b^NRT: nicotine replacement therapy.

^c^NYS: New York State.

### Recruitment and Eligibility

Targeted recruitment occurs through the posting of flyers in community-based organizations and the submission of web-based advertisements (ie, Craigslist and RecruitMe), as well as through snowball sampling [23] of study participants. Potential participants are instructed to call our office line or contact the study email to participate in screening procedures over the phone with a study team member. The eligibility criteria for this study are presented in Textbox 1.

**Inclusion and exclusion criteria.**

**Inclusion criteria**

- People living with HIV confirmed through medical records or pill bottles for antiretroviral therapy (ART) medications.
- Aged 18 years or older.
- Able to understand and read English.
- Current smoker (smokes at least five cigarettes per day for the past 30 days).
- Owns an Android smartphone.
- Not pregnant or breastfeeding (due to contraindications for nicotine replacement therapy [NRT]).
- Permanent contact information.
- Interested in quitting smoking within 30 days.
- Exhaled carbon monoxide (eCO) ≥5 ppm (parts per million) at baseline.

**Exclusion criteria**

- Use of tobacco products other than cigarettes (ie, vapes, electronic cigarettes [e-cigarettes], cigars, piped tobacco, chew, and snuff).
- Planning to move within 3 months of enrollment.
- Alcohol dependence as measured through the Alcohol Use Disorders Identification Test-Concise (AUDIT-C).
- Positive history of a medical condition that precludes nicotine patch use.
- Current use of NRT or other smoking cessation medications (eg, Chantix or Zyban).
- Current enrollment in another smoking cessation program
- A household member is also participating in the Sense2Quit study, as it can lead to study contamination [24].

#### Sample Size Calculation

This study is a pilot study, and therefore it is not powered to detect efficacy. In this pilot study, we propose a total sample of 60 (30 per arm group) with a 20% expected attrition rate. Therefore, this study is not powered to detect a significant difference between study arms. Instead, we estimated the precision of proportion estimates (cessation rates) for the given sample size (30 per arm). For the proposed sample size of 30 per group, we will have at least an 80% probability that the SE of the cessation rate estimates are ≤7%.

#### Randomization

Eligibility is confirmed at the in-person baseline visit. Participants confirm their HIV status by showing the study team their ART prescription or other medical records that provide HIV status. Smoking status is confirmed by the participant having an exhaled carbon monoxide (eCO) level of ≥5 ppm (parts per million). Upon confirmation of eligibility, participants complete the informed consent process and are randomized to the study group using a computer random number generator in Research Electronic Data Capture (REDCap). Study personnel are blinded to randomization before enrollment. Sex (assigned at birth)-stratified randomization is achieved through random-permuted blocks.

### Description of the Intervention: Sense2Quit App

**Figure 1:**
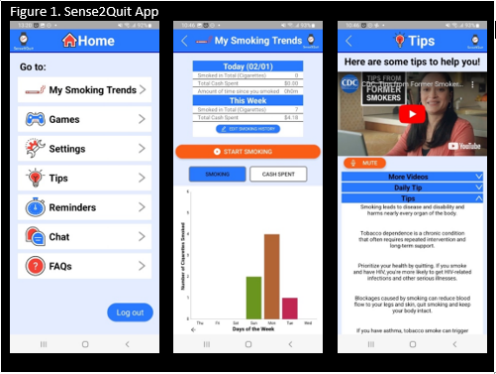
Sense2Quit app.

The Sense2Quit app (Figure 1) is a mobile app connected through Bluetooth to a smartwatch that tracks smoking gestures and distinguishes them from other everyday movements such as eating, drinking, etc. The ISR framework guided the development of the app and included an iterative process [14,15] where both people living with HIV, who were current or former smokers, and usability experts helped design, evaluate, and refine the Sense2Quit app. Findings from the formative work were used to inform the final Sense2Quit app, which was used in the RCT [14,15].

In the Sense2Quit app, participants can view their smoking trends, which are recorded through their use of the smartwatch, including how often or how much they smoke and the amount of money that they are spending on cigarettes, watch videos with quitting tips, information, and distractions, play Pac-Man and Tedroid (a game similar to Tetris [25]), set reminders, and communicate with a study team member.

Following enrollment in the study, participants who are randomized to the intervention arm receive a smartwatch and access to the Sense2Quit app. Participants are assigned a username (their study ID, SQ, followed by a number assigned chronologically) and can create a password for themselves. A study team member familiarizes the participant with the app, highlighting each component and feature. Participants can send a chat or call the study office whenever they have a question about the app or watch.

Study staff will encourage participants to wear their smartwatch on a daily basis so that it accurately captures smoking data. All smoking data will be displayed on the “My Smoking Trends” page of the app in the form of a graph. If the smartwatch incorrectly detects smoking, participants can click “Edit Smoking History” to update their smoking data for the previous week. Participants will be informed of the potential benefits of using the “Games,” “Tips,” and “Reminders” pages of the app, such as distracting themselves to overcome an urge to smoke by playing Pac-Man or Tetroid or watching a video. Additionally, tailored reminders can be set to overcome daily urges such as those surrounding morning coffee or a meal. Study staff will check in with participants throughout the study about any issues that arise while using the app, and participants will come into the office for a tech appointment when necessary.

### Study Assessments

Participants are enrolled in person at the research office. After enrollment, participants in both arms complete study assessments at their baseline, 4-week, and 12-week follow-up visits, for a total of 3 study visits. Surveys are administered through Qualtrics (Qualtrics Experience Management [XM]) and gather information about participant demographics [26], tobacco use history, tobacco cessation (primary outcome: self-report of whether or not they have smoked or used other tobacco products in the last 7 days; number of quit attempts), illicit substance and alcohol use [27,28], predictors of tobacco cessation [29,30], cravings and withdrawal [31], psychosocial factors [32-34], markers of HIV/AIDS immune status [35], and pharmacotherapy use. All instruments used in the surveys have consistently demonstrated high reliability and validity [26-37]. The Health Information Technology Usability Evaluation Scale (Health-ITUES) and Post-Study System Usability Questionnaire (PSSUQ) will be used on the 12-week follow-up assessment to help us determine the acceptability of the intervention. We will ask participants in both arms how useful each of the intervention components was using the PSSUQ with a 7-point Likert scale (1=strongly agree to 7=strongly disagree) [37] and Health-ITUES with a 5-point Likert scale (1=strongly disagree to 5=strongly agree) [38].

### Primary Outcome

The primary outcome is the percentage of participants with a biochemically verified 7-day point prevalence of smoking or tobacco abstinence. The 7-day point prevalence of abstinence is calculated as the percentage of participants who reported no smoking or tobacco use in the 7 days before their 12-week follow-up visits (self-report on survey whether or not they have smoked cigarettes, even just a puff, or used other forms of tobacco in the past 7 days), which is then biochemically verified by eCO collected at 12 weeks through a breathalyzer (Micro+TM basic Smokerlyzer, coVita). Participants with eCO levels <5 ppm will be classified as abstinent, while participants with eCO levels ≥5 ppm will be classified as not abstinent. Although we exclude participants who report use of tobacco products other than cigarettes at baseline, we anticipate that some participants may use other tobacco products during the study period. Thus, abstinence will be defined as having refrained from using not only cigarettes but also all tobacco products.

### Statistical Analysis

This is a pilot study to assess feasibility, acceptability, and preliminary efficacy. To determine feasibility, we will examine retention rates, eligibility criteria, recruitment and enrollment rates, missing data, and study measures. Descriptive statistics will be used for the analysis of demographic characteristics. The rate of quit attempts will be calculated as the proportion of smokers who made a quit attempt (defined as no smoking for at least a 24-hour period); 7-day point prevalence abstinence will be calculated as the proportion of smokers who have not smoked or used tobacco products in the 7 days before the study visit (as reported in the 4- and 12-week follow-up surveys) and verified by an eCO level of <5 ppm. We will provide point estimates and corresponding CIs of these measures for each arm. Because of the small sample sizes, we will obtain Clopper-Pearson exact CIs based on the binomial distribution. Given the small sample size, the purpose of the arms is to monitor for unexpected, gross differences between the 2 groups. The preliminary efficacy of the intervention will be based on 7-day point prevalence abstinence at the 12-week follow-up survey. Participants lost to follow-up will be included as smokers because we will be missing follow-up data and cannot determine whether or not the intervention was effective. Since this is a pilot study, we will be focusing on the acceptability, demand, and limited efficacy testing of the intervention. Acceptability will be assessed by evaluating how the participants react to the intervention (using PSSUQ and Health-ITUES). The demand for the intervention will be assessed by gathering data on estimated use. The limited-**efficacy testing** will be conducted on a convenience sample with a short follow-up period and limited statistical power [39].

## Results

Enrollment of study participants began in March 2023 and is expected to end in October 2023. All data collection is expected to be completed by the end of January 2024. Results from this pilot study will be disseminated to the research community following the completion of all data collection.

## Discussion

### Overview

Our planned study will test the effectiveness of the Sense2Quit app for smoking cessation among people living with HIV in New York City. This RCT will assess the difference in outcomes between the control arm, receiving smoking cessation counseling and Quitline referral, and the intervention arm, receiving smoking cessation counseling, an 8-week supply of combination NRT, and a smartwatch linked to the Sense2Quit app. This study plans to address a gap in the literature surrounding mHealth for people living with HIV who smoke.

Smartwatch technology has been developed and tested in interventional smoking cessation studies within the past decade, yet study samples have been small, and more testing is required to improve the accuracy of smoking detection [40-43]. Similarly, mHealth has been used for smoking cessation and has been found to be effective in the short term [44-51], with little evidence of efficacy in the long term [52]. A systematic review of quit-smoking apps in Android’s Play Store and Apple’s App Store found high rates of existing irrelevant or nonfunctioning apps and few apps with evidence-based support [53]. Thus, despite the large volume of apps to help users quit smoking that are currently on the market, few are likely to be effective.

Smoking cessation mHealth studies among people living with HIV are limited [12]. Tobacco cessation studies targeting people living with HIV have mostly consisted of automated messaging systems to intervene with smoking habits, motivational interviewing, and provision of pharmacotherapy [11,54-56]. In most cases, mHealth interventions for people living with HIV who smoke have been found to be more effective than face-to-face interventions [11], yet there is a need for more research on mHealth interventions which include interactive components such as communication with the study team or smoking cessation counseling [57]. Because people living with HIV are likely to have a shorter life span if they are chronic smokers [3], further research on effective quitting tools for people living with HIV is needed.

Although we found that people living with HIV in our focus groups were highly motivated to quit smoking due to health concerns, they identified needing additional interventions and support to successfully quit smoking amid everyday challenges and struggles [14]. With more than 350,000 apps currently on the market, mHealth technology is becoming widely known and used among people of all ages, races, and ethnicities [58,59]. mHealth technology is a promising tool across socioeconomic groups, as roughly 76% of low-income households (earning US $30,000 or less yearly) in the United States own a smartphone and indicate that their smartphone is their primary source of internet access [60,61]. Consequently, mHealth technology is a tool that is used in people’s everyday lives and therefore can help with promoting health behavior change. This study will help us determine the potential of the Sense2Quit app to help people living with HIV quit smoking cigarettes. To our knowledge, the Sense2Quit study is the only existing study to use mHealth combined with a smartwatch to guide people living with HIV through their quitting process in New York City.

### Limitations

This pilot study plans to recruit 60 people living with HIV in New York City who smoke cigarettes. As a result, the population may be too small to make generalizations about effective tobacco cessation techniques among people living with HIV in the short or long term. A second limitation is that although we will exclude people who use other tobacco products, such as cigars and vapes, we do not exclude people who may smoke other drugs, such as marijuana. This may result in incorrect smoking data for some participants. Participants will be instructed, however, on how to edit their smoking log in the app if incorrect data is shown.

### Conclusions

The Sense2Quit study leverages mHealth so that it can help smokers improve their efforts at smoking cessation. Our research has the potential to not only increase quitting rates among people living with HIV who may need a prolonged, tailored intervention but also inform further development of mHealth for people living with HIV. This mHealth intervention also has the potential to address health inequities among people of different socioeconomic groups, as the majority of people in the United States own a smartphone and have access to apps. Finally, this study will contribute significant findings to the greater mHealth research community, providing evidence as to how mHealth should be developed and tested among the target population.

## Abbreviations

ART: antiretroviral therapy
eCO: exhaled carbon monoxide.
Health-ITUES: Health Information Technology Usability Evaluation Scale
HIPAA: Health Insurance Portability and Accountability Act
ISR: Information Systems Research
mHealth: mobile health
NRT: nicotine replacement therapy
NYS: New York State
ppm: parts per million
PSSUQ: Post-Study System Usability Questionnaire
RCT: randomized controlled trial
REDCap: Research Electronic Data Capture

## Data Availability

The data sets generated during this study are available from the corresponding author on reasonable request.
